# TRPC5 involvement in NTG-induced migraine-like central sensitization in mice: a study based on the PI3K-Akt/P2X7R-IL-1β signaling axis

**DOI:** 10.3389/fimmu.2026.2018949

**Published:** 2026-10-29

**Authors:** Jing Lu, Chenlu Zhu

**Affiliations:** 1 Department of Clinical Laboratory Medicine, Shandong Medicine and Health Key Laboratory of Laboratory Medicine, The First Affiliated Hospital of Shandong First Medical University & Shandong Provincial Qianfoshan Hospital, Jinan, Shandong, China; 2 Department of Neurology, The First Affiliated Hospital of Shandong First Medical University & Shandong Provincial Qianfoshan Hospital, Jinan, China

**Keywords:** central sensitization, chronic migraine, neuroinflammation, PI3K-Akt signaling, TRPC5

## Abstract

**Background:**

Central sensitization is the core pathological mechanism driving the occurrence and chronification of chronic migraine (CM). Transient Receptor Potential Canonical 5 (TRPC5) channel is critically involved in the modulation of multiple pain disorders and central sensitization. However, the specific regulatory role and precise molecular mechanism of TRPC5 in the progression of acute and chronic migraine remain unclear. This study aimed to explore the potential function of TRPC5 and its downstream signaling cascade in nitroglycerin (NTG)-induced migraine-like central sensitization, so as to provide novel preclinical evidence for migraine pathogenesis and targeted intervention.

**Methods:**

A repetitive NTG intraperitoneal injection protocol was applied to establish a mouse model mimicking acute migraine nociceptive responses and chronic migraine central sensitization. Migraine-like behavioral phenotypes, including mechanical allodynia, thermal hyperalgesia and light-aversive behavior, were evaluated at both acute and chronic modeling stages. qRT-PCR and Western blot were performed to detect TRPC5 mRNA and protein expression in the primary somatosensory (SSP) cortex. Immunofluorescence staining was used to verify the neuronal localization of upregulated TRPC5 and assess the activation status of cortical neurons and microglia. Pharmacological interventions with TRPC5 selective antagonist AC1903 and phosphoinositide 3-kinase-protein kinase B (PI3K-Akt) inhibitor LY294002 were applied to validate the regulatory relationship among TRPC5, PI3K-Akt signaling and purinergic receptor P2X7/interleukin-1 beta (P2X7R/IL-1β)inflammatory axis.

**Results:**

Repeated NTG challenge induced robust migraine-like behaviors, prominent neuronal hyperactivation and sustained central sensitization within the SSP cortex. TRPC5 mRNA and protein were markedly increased mainly in SSP cortical neurons. NTG also triggered microglial overactivation and upregulated P2X7R and IL-1β. AC1903-mediated TRPC5 inhibition alleviated acute and chronic migraine-like phenotypes, blunted neuronal and microglial activation, suppressed PI3K-Akt overactivation and reduced P2X7R/IL-1β expression. LY294002 blockade of PI3K-Akt recapitulated these protective effects, attenuating sensory hypersensitivity and neuroinflammation.

**Conclusion:**

TRPC5 mediates NTG-induced migraine central sensitization. Neuronal TRPC5 upregulation activates PI3K-Akt signaling, inducing neuronal hyperexcitability, microglial overactivation and P2X7R/IL-1β neuroinflammation. This TRPC5-dependent axis controls acute nociception and chronic migraine progression. Our findings uncover neuron-microglia crosstalk and support TRPC5 as a preclinical therapeutic target for migraine.

## 1 Introduction

Migraine is a disabling neurological disorder characterized by recurrent moderate-to-severe throbbing headache, mostly unilateral, frequently accompanied by nausea, vomiting, and heightened responsiveness to light and sound stimuli (1, 2). Epidemiological data indicate that approximately 3% of patients with episodic migraine progress to chronic migraine each year (3). Following progression to chronic migraine, patients exhibit substantially elevated risks of anxiety, depression, sleep disturbance, stroke and cardiovascular-related mortality (4, 5), impairing the quality of life of nearly one-billion individuals worldwide (6). Cumulative evidence suggests that migraine arises primarily from intrinsic functional and structural abnormalities within the central nervous system, rather than simple secondary peripheral injury (7). Aberrant neuronal hyperexcitability and maladaptive synaptic plasticity initiate and sustain central and peripheral nociceptive sensitization, participating in the pathophysiological processes underlying migraine onset and progression (8–10).

Accumulating evidence highlights the critical role of neuron-glia crosstalk in migraine chronification. Reciprocally activated neurons and glial cells release abundant inflammatory mediators and neuropeptides, further sustaining neuronal hyperexcitability and forming a self-amplifying pathological loop that may drive recurrent migraine attacks and transition toward chronicity. Migraine-associated central sensitization involves aberrant neuronal activation across multiple brain regions. The trigeminovascular system constitutes the core functional pathway mediating migraine nociception, with the trigeminal ganglion (TG) and spinal trigeminal nucleus caudalis (Sp5C/TNC) serving as key components of this pathway (11–13). Beyond brainstem and peripheral trigeminal structures, the cerebral cortex participates in nociceptive discrimination, integration and nociceptive plasticity remodeling. The primary somatosensory cortex (SSP) contributes to nociceptive information processing. Preclinical animal studies have suggested that this brain region undergoes pathological changes linked to migraine-related central sensitization; therefore, the SSP cortex was selected as the primary target region for the present study.

Transient receptor potential canonical 5 (TRPC5) is a non-selective cation channel belonging to the TRP superfamily. The human TRPC5 gene locates on chromosome X and encodes a channel protein containing six transmembrane domains. Its unique biophysical properties enable TRPC5 to participate in diverse physiological and pathological processes. Structurally, TRPC5 possesses a voltage-sensing domain that senses alterations in intra- and extracellular microenvironments to modulate channel gating and opening kinetics (14). TRPC5 can be activated by multiple upstream stimuli including membrane depolarization, mechanical stimulation and endogenous signaling molecules (15). Furthermore, the intracellular C-terminal domain of TRPC5 interacts extensively with downstream signaling molecules for functional coupling, regulating multiple intracellular signaling cascades and exerting essential biological functions in the central nervous system and cardiovascular system (16).

TRPC5 is widely distributed in multiple pain-related tissues and cells, including the cerebral cortex, hypothalamus, TG, dorsal root ganglia (DRG), retinal neurons, odontoblasts and synoviocytes, implying its potential involvement in pain modulation (17). Existing studies have documented TRPC5 functions in diverse pain models. For instance, upregulated TRPC5 contributes to opioid-induced hyperalgesia and mechanical allodynia in pulpitis pain (18). Notably, TRPC5 is highly expressed in key brain regions implicated in migraine-related central sensitization: cerebral cortex, hypothalamus, midbrain, medulla oblongata and TG, with approximately 30% of trigeminal ganglion neurons being TRPC5-positive (19). It should be noted that reference (19) mainly focuses on TG; the cellular distribution of cortical TRPC5 across neurons and glial cells remains incompletely clarified. Despite clues from expression profiles and pain-related investigations, the exact function and detailed molecular mechanisms of TRPC5 in migraine pathogenesis remain to be elucidated.

The PI3K-Akt signaling cascade represents a core pathway governing central sensitization, modulating neuronal plasticity, morphological remodeling and synaptic transmission efficiency, and amplifying nociceptive signal integration and persistence (20, 21). Under pathological nociceptive insults, excessive PI3K-Akt activation elicits robust neuroinflammatory responses and promotes secretion of pro-inflammatory mediators such as IL-1β and TNF-α (22). These pro-inflammatory cytokines further trigger microglial over-activation, enhance inflammatory transcriptional activity and establish positive-feedback loops that exacerbate neuroinflammation and central sensitization (23). Given that both TRPC5 and PI3K-Akt participate in pain modulation and central sensitization, we hypothesized that TRPC5 might modulate PI3K-Akt-dependent neuroinflammatory cascades and thereby contribute to migraine-like pathological alterations.

The working hypothesis of this study is that TRPC5 modulates PI3K-Akt signaling activation and subsequent neuroinflammatory responses to participate in NTG-evoked migraine-associated central sensitization. It should be clarified that repeated NTG administration in animals only recapitulates migraine-like behavioral phenotypes and cannot fully recapitulate the natural clinical progression from episodic to chronic migraine in human patients. Furthermore, this study primarily relies on pharmacological tools for intervention, which carry potential off-target risks. Using the well-established NTG-induced migraine mouse model, we systematically explored the effects of TRPC5 on acute migraine-like behaviors and persistent central sensitization phenotypes. We also analyzed potential associations among PI3K-Akt-mediated neuron-glia crosstalk and the P2X7R/IL-1β inflammatory axis. This study aims to provide novel experimental insights into cortical central sensitization in migraine and identify potential pre-clinical targets for migraine intervention.

## 2 Materials and methods

### 2.1 Experimental animals

Male C57BL/6 mice aged 8-12 weeks weighing 20-25 g were purchased from Jinan Pengyue Experimental Animal Breeding Co., Ltd. All mice were housed under specific-pathogen-free (SPF) standardized conditions: temperature 22 ± 2 °C, relative humidity 50 ± 5%, 12 h light-dark cycle, with ad libitum access to food and water. Mice were acclimatized for 2 weeks prior to formal experiments to eliminate confounding environmental stress on behavioral and molecular measurements.

All animal experimental protocols were reviewed and approved by the Animal Ethics Committee of the First Affiliated Hospital of Shandong First Medical University. Experimental procedures strictly complied with the ethical guidelines for pain research in conscious animals issued by the International Association for the Study of Pain (IASP) and the ARRIVE reporting guidelines for animal experiments. Animal grouping, experimental manipulations, behavioral testing, molecular assays and image analysis were performed under double-blind conditions; experimenters and analysts remained unaware of group assignments throughout the study.

Simple randomization was applied for animal allocation. Sample size was estimated based on effect sizes from preliminary experiments. Clear inclusion and exclusion criteria were predefined; the number and reasons for animal exclusion were fully documented. Independent cohorts of mice were used for behavioral assays, immunofluorescence staining, qRT-PCR and Western blot to avoid data bias caused by repeated testing on the same animals. Rules for animal assignment across different experiments were explicitly defined.

### 2.2 Experimental drugs and reagent preparation

Nitroglycerin (NTG) was freshly prepared in 0.9% sterile normal saline at a stable working concentration. The administered dose was 10 mg/kg with a standardized intraperitoneal injection volume of 10 mL/kg across all groups. The TRPC5-selective inhibitor AC1903 (Cat. No. HY-122051, MCE, purity ≥99.69%) was prepared following the manufacturer’s protocol and published paper (24). A high-concentration stock solution was prepared in anhydrous DMSO, aliquoted, and stored at −80 °C protected from light. Before use, the stock was diluted into working solution using a mixed solvent system (10% DMSO + 40% PEG300 + 5% Tween-80 + 45% sterile normal saline). Complete solubilization was achieved by vigorous vortexing combined with short-term ice-bath sonication. All working solutions were freshly prepared. An equal-volume vehicle control with identical solvent composition was included to exclude solvent-derived interference with pain thresholds and neuroinflammation.

The PI3K inhibitor LY294002 (MCE, HY-10108) was used for mechanistic validation. LY294002 was intraperitoneally injected at 20 mg/kg with an injection volume of 10 mL/kg, dissolved in sterile normal saline, administered 30 min before each NTG modeling session, which was modified little and relied on published literature (25).

### 2.3 Model establishment and experimental groups

A repeated intraperitoneal NTG injection protocol was adopted to establish the mouse model of migraine-like central sensitization, which reliably reproduces persistent mechanical allodynia and central sensitization phenotypes. Modeling lasted for 9 days; NTG (10 mg/kg, i.p.) was administered every other day on day 1, 3, 5, 7 and 9 to induce persistent mechanical nociceptive hypersensitivity and neuroinflammatory alterations.

Timing definitions were strictly unified: pain thresholds measured 2 h before the final NTG injection represented persistent basal nociceptive hypersensitivity (chronic migraine-like phenotype). Behavioral and molecular indices detected 2 h post-NTG injection reflected acute migraine-like nociceptive responses. This experimental model recapitulates only migraine-like behaviors and pathological central sensitization in mice and cannot fully replicate the natural clinical progression from episodic to chronic migraine in humans.

The dedicated NTG vehicle (6% propylene glycol + 6% ethanol in saline) and pure 0.9% normal saline exerted no significant effects on mechanical withdrawal thresholds in preliminary tests; accordingly, 0.9% normal saline served as the blank control throughout this study. Four experimental groups were established: ① Saline group; ② Saline + AC1903 group; ③ NTG model group; ④ NTG + AC1903 group; ⑤ NTG+LY294002. Eight mice per group were used for behavioral testing; independent animal cohorts were used for molecular and histological assays.

### 2.4 Behavioral assays

All behavioral tests were performed between 09:00 and 15:00 under stable circadian conditions. Mice were habituated to the testing room for 30 min before testing to reduce stress-related confounders. Testing equipment was wiped with 75% ethanol after each mouse and each trial to eliminate odor and excrement residues and prevent cross-interference. A fixed testing sequence was followed: von-Frey filament test → hot-plate test → light-dark box test. Behavioral data were collected at 2 h before and 2 h after modeling drug administration.

#### 2.4.1 Mechanical nociceptive hypersensitivity (von-Frey Filament assay)

Graded von-Frey filaments ranging from 0.008-4 g were applied. The Dixon up-down method, Chaplan’s classic protocol and Christensen’s modified algorithm were combined to determine 50% paw-withdrawal thresholds. Mechanical thresholds were measured at both hind-paw plantar surfaces and periorbital regions. Periorbital thresholds were used to evaluate migraine-specific cephalic nociceptive hypersensitivity, while hind-paw thresholds assessed generalized central sensitization. A positive response (X) was defined as paw withdrawal, head flicking or avoidance behavior; absence of obvious behavioral changes was scored as negative (O). All original X/O response sequences and final filament ranks were imported into the online von-Frey threshold analysis platform (https://bioapps.shinyapps.io/von_frey_app/).

Target-force recognition and the exact-delta algorithm were applied to calculate 50% mechanical withdrawal thresholds for improved accuracy. This readout reflects stimulus-evoked withdrawal behaviors in mice and does not directly equate to subjective pain perception in humans. Terms such as mechanical withdrawal threshold reduction and mechanical nociceptive hypersensitivity-like behavior were consistently adopted; definitive terms such as allodynia were used cautiously.

#### 2.4.2 Hot-plate test

A thermostatically controlled hot-plate instrument maintained at 55 ± 0.5 °C was used to measure thermal nociceptive latency for assessment of central nociceptive sensitization. Mice were gently placed onto the hot-plate surface. Latencies to hind-paw withdrawal or paw-licking were recorded, with a maximum cutoff time of 30 s to prevent thermal skin injury. Each mouse was tested twice with a 15-min inter-trial interval; the mean value of two measurements was taken for statistical analysis. Short-term repeated thermal stimulation does not produce persistent tissue damage and reliably captures baseline thermal nociception and sensitization shifts, as validated in previous studies. This assay evaluates thermal nociceptive sensitivity and was not conflated with mechanical nociceptive indices.

#### 2.4.3 Light-aversive behavior in light-dark box

The light-dark box apparatus measured 50 cm × 25 cm × 30 cm with an 8 cm × 8 cm interconnecting aperture between compartments. The light compartment was illuminated at 300 lx; the dark compartment maintained 0 lx, with overhead light calibrated at the animal activity plane. At assay onset, each mouse was placed into the dark compartment facing inward without prior pre-exposure habituation. The ANY-maze behavioral tracking software recorded animal trajectories continuously for 10 min; primary readout was time spent in the light compartment. Given the involvement of TRPC5 in anxiety- and fear-related regulation, prolonged light-compartment residence time cannot be solely attributed to relief from migraine-related photophobia. The objective description “light-aversive-like behavior” was consistently used throughout the manuscript to avoid over-interpretation of causal relationships.

### 2.5 Brain tissue collection and sample preparation

All animals were sacrificed 12 h after the final drug or modeling intervention. This time-point was selected according to reference data on c-Fos expression windows and pro-inflammatory cytokine peak levels and kept consistent across all experiments. Mice were deeply anesthetized with 3% isoflurane, perfused transcardially with ice-cold 0.01 M PBS (pH 7.4) to clear intravascular blood. Brains were rapidly dissected and the SSP cortex was precisely isolated. All molecular and histological analyses in this study targeted the SSP cortex. Samples for molecular experiments were immediately snap-frozen in liquid nitrogen and stored at −80 °C. For immunofluorescence, tissues were perfusion-fixed in 4% paraformaldehyde, post-fixed overnight, and dehydrated sequentially in 20% and 30% sucrose for 24 h each prior to sectioning.

### 2.6 Western blot

SSP cortical tissues were homogenized on ice in RIPA lysis buffer supplemented with protease and phosphatase inhibitors. Total protein supernatants were collected after high-speed centrifugation. Protein concentrations were quantified using the BCA assay. Equal amounts of protein (30 μg per lane) were separated by 12% SDS-PAGE and electro-transferred onto PVDF membranes. Membranes were blocked with 5% non-fat milk for 30 min at room temperature and thoroughly washed with PBST.

Primary antibodies were incubated overnight at 4 °C with complete experimental information as follows: TRPC5 (1:200, Proteintech, 25890-1-AP, RRID: AB_2880285); Phospho-PI3K (1:1000, CST, 4228S, RRID: AB_2262433); Total-PI3K (1:1000, CST, 4257S, RRID: AB_2234321); Phospho-Akt (1:2000, CST, 4060S, RRID: AB_2315049); Total-Akt (1:1000, CST, 4691S, RRID: AB_10001375); P2X7R (1:1000, Abcam, ab259942, RRID: AB_2922041); IL-1β (1:1000, Abcam, ab254360, RRID: AB_2910747); β-actin (1:5000, Abcam, ab8226, RRID: AB_449644). HRP-conjugated goat anti-rabbit/goat anti-mouse secondary antibodies (1:10000, Jackson ImmunoResearch) were incubated for 1 h at room temperature. Bands were visualized using ECL chemiluminescent substrate protected from light, and raw images were acquired on a Bio-Rad imaging system. Densitometric quantification was performed with ImageJ software. Phosphorylated protein signals were normalized to their corresponding total protein levels; target protein abundances were normalized against appropriate internal reference proteins.

### 2.7 qRT-PCR

Total RNA was extracted from SSP cortex using Trizol reagent. Complementary DNA was synthesized via reverse-transcription kits. Amplification was performed on a QuantStudio 6 Flex real-time PCR instrument using SYBR Green fluorescent dye. Thermal cycling parameters: initial denaturation at 95 °C for 20 s; 40 cycles of 95 °C for 15 s, 60 °C for 30 s, 72 °C for 30 s. Melting-curve analysis verified primer specificity. All primers were designed using NCBI Primer-BLAST with amplicon lengths of 80-150 bp. Primer sequences were annotated with 5′-3′ orientation, GenBank accession numbers and amplification efficiency. The relative mRNA expression levels of target genes were normalized to GAPDH and calculated using the 2^−^ΔΔCt method. The gene primers are shown below in **Table 1**.

**Table 1:** Gene primer sequence.

Gene	Forward primer sequence	Reverse primer sequence
TRPC5	AATGTCATCTCCCTGG TAGTG	AGCATGATCGGCAAT AAGC
P2X7R	TTTATCCCTGGTGCA AGTG	CTTTAATGTCGGCTT TGGC
IL-1ß	AAGAACCTATCTTCTTCG ACAC	GAGAACACCACTTGT TGCT

### 2.8 Immunofluorescence co-localization staining

Dehydrated brain tissues were sectioned at 40 μm coronal slices using a Leica VT1200S vibratome, and slices containing the SSP cortex were selected for staining. Slices were permeabilized with 0.3% Triton X-100 at room temperature and blocked with 5% normal donkey serum to suppress non-specific binding. Primary antibodies against TRPC5, Iba1 and c-Fos (all diluted 1:500) were incubated for 48 h at 4 °C. After thorough PBST washing, Alexa Fluor-conjugated secondary antibodies (1:1000) were applied for 2 h at room temperature under light-protected conditions. Nuclei were counter-stained with DAPI.

Z-stack scanning was performed on a Nikon A1R confocal microscope with a step size of 1 μm. Images were captured using NIS-Elements software. The ImageJ JACoP plugin was used for co-localization analysis; Pearson correlation coefficients were reported.

### 2.9 Statistical analysis

All data were expressed as mean ± SEM. Error bars in figures represent SEM; individual raw data points were overlaid on all graphs to improve data transparency. Statistical analyses were conducted using GraphPad Prism 8.0 and SPSS 22.0. The Kolmogorov-Smirnov test was used to verify normal distribution of datasets. Two-tailed Student’s t-test was used for comparisons between two independent groups. One-way ANOVA followed by Tukey’s post-hoc test was applied for multiple-group comparisons. Repeated-measures ANOVA (RM-ANOVA) with Tukey’s post-hoc test was used for longitudinal behavioral measurements within the same animal. All statistical analyses followed the blinding principle; each individual mouse was treated as an independent statistical unit. P < 0.05 was defined as statistically significant.

## 3 Results

### 3.1 NTG-induced migraine-like behaviors and up-regulation of TRPC5 in the SSP cortex

To explore the potential role of TRPC5 in migraine-like central sensitization, a mouse migraine-like model was established by repeated intraperitoneal NTG injection (**Figure 1A**). Compared with the saline control group, NTG-treated mice exhibited significantly decreased mechanical withdrawal thresholds under both persistent basal nociceptive hypersensitivity and post-injection acute nociceptive challenge conditions, indicating successful induction of migraine-like nociceptive hypersensitivity (**Figures 1B, C**). Meanwhile, NTG markedly increased the number of c-Fos-positive cells within the SSP cortex, indicating prominent cellular hyperactivation in this brain region (**Figure 1D**).

**Figure 1:**
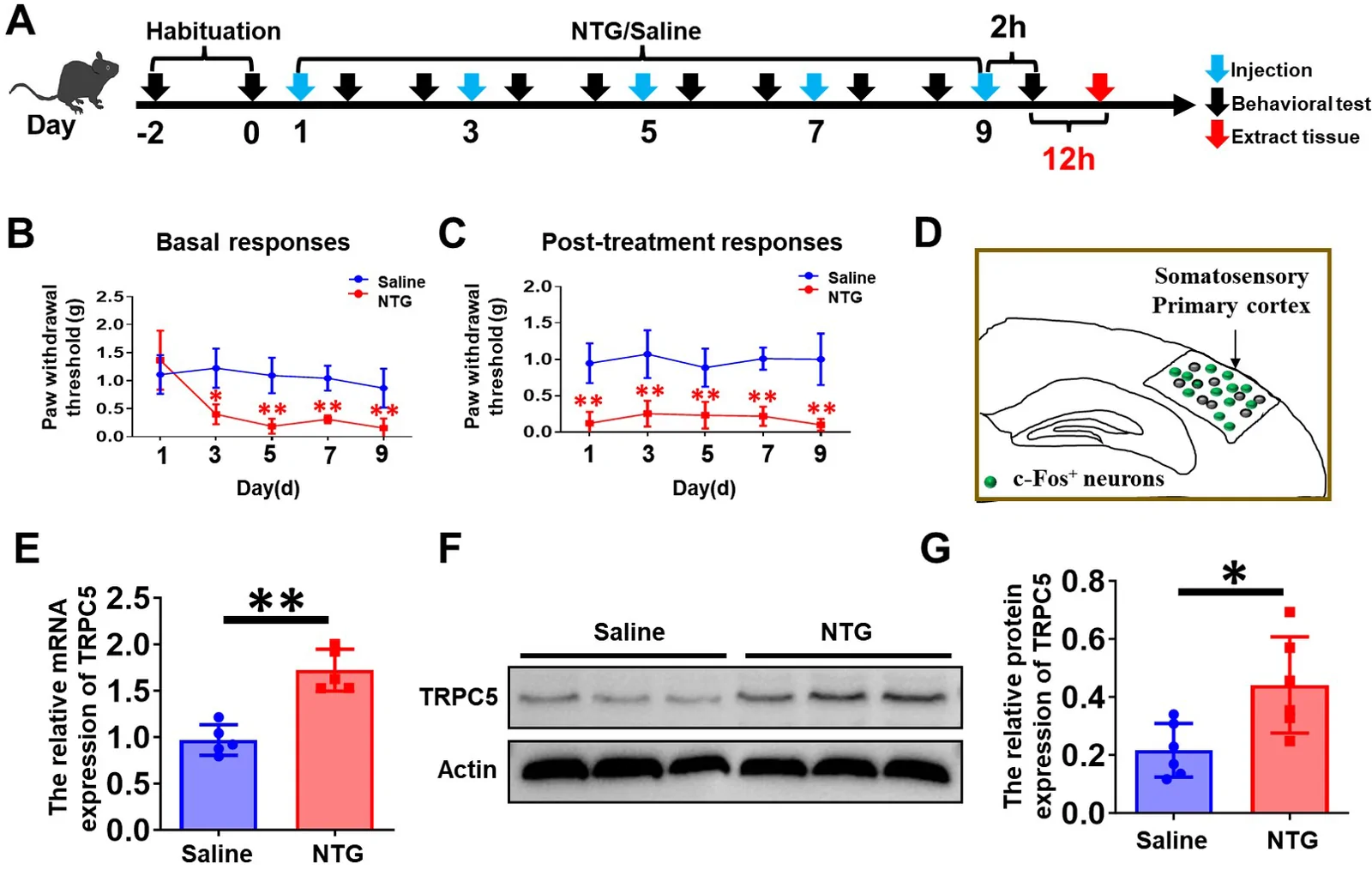
Chronic NTG exposure upregulates TRPC5 expression in the SSP cortex of migraine model mice. **(A)** Schematic illustration of the establishment procedure for the NTG-induced migraine mouse model. **(B, C)** Migraine-like mice exhibited significantly reduced pain thresholds under chronic **(B)** and acute **(C)** pain conditions. Statistical analyses were performed by two-way ANOVA and Tukey *post hoc* test; *P<0.05, **P<0.01 (n=8 mice per group). **(D)** Schematic diagram showing an increased number of c-Fos-positive activated neurons in the primary somatosensory (SSP) cortex during migraine states. **(E)** TRPC5 mRNA expression was markedly elevated in the SSP cortex of NTG-treated migraine mice. **(F, G)** Western blot results and quantitative analysis verified that NTG treatment significantly increased TRPC5 protein levels in the SSP cortex of migraine mice. Data are presented as mean ± SEM; n=4 per group; *P<0.05 compared to the Saline group. Data are presented as mean ± SEM. Behavioral tests: n = 8 per group; statistical analyses were performed using two-way ANOVA with Tukey *post hoc* tests; molecular detection: n = 5 per group. Statistical analysis was performed using a two-tailed independent samples t-test. *P<0.05, **P<0.01 versus the Saline group.

Molecular assays demonstrated that NTG intervention significantly elevated both mRNA and protein levels of TRPC5 in the SSP cortex (**Figures 1E–G**). Collectively, repeated NTG exposure stably triggered migraine-like behaviors and cortical central sensitization phenotypes accompanied by increased TRPC5 expression in the SSP cortex. Baseline pain thresholds measured before drug administration represented persistent nociceptive hypersensitivity, whereas behavioral and molecular indices acquired 2 h after NTG injection reflected acute nociceptive responses.

### 3.2 Neuronal TRPC5 up-regulation in the SSP cortex following NTG exposure

Immunofluorescence co-localization staining for TRPC5 and the neuronal marker NeuN was performed to characterize TRPC5 distribution within the SSP cortex (**Figure 2A**). The number of TRPC5-positive neurons and the proportion of TRPC5-positive neurons among total NeuN-positive neurons were markedly higher in the NTG group (**Figures 2B, C**). These findings suggest that NTG-driven TRPC5 up-regulation occurs predominantly in SSP cortical neurons. It should be noted that glia-specific co-localization experiments were not performed in this study; thus, changes in glial TRPC5 expression cannot be fully excluded. Therefore, we can only infer neuronal TRPC5 up-regulation rather than concluding exclusive neuronal expression of TRPC5.

**Figure 2:**
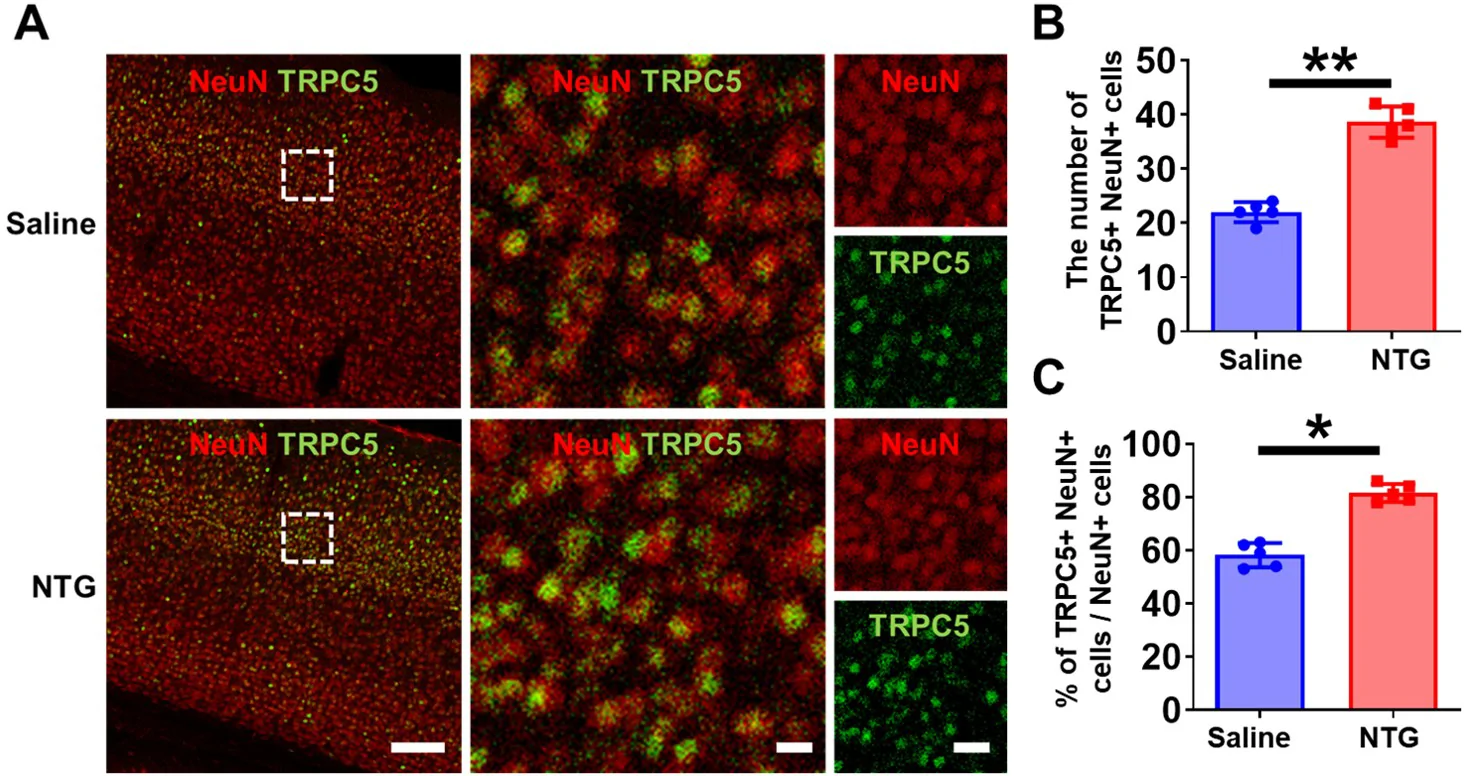
Neuronal TRPC5 expression is upregulated in the SSP region following NTG treatment. **(A)** Representative immunofluorescence colocalization staining for TRPC5 and NeuN within the SSP region in the Saline and NTG groups. The left panel was captured at a scale bar of 100 μm, and the magnified view within the dashed white box on the right corresponds to a scale bar of 10 μm. **(B, C)** Quantitative quantification of TRPC5^+^NeuN^+^ neuronal counts **(B)** and the proportion of TRPC5^+^NeuN^+^ neurons relative to total NeuN^+^ neurons **(C)** within a 360 × 360 μm² field of view per brain section per mouse. More TRPC5-expressing neurons were detected in the NTG group relative to the Saline group in the SSP region. Data are expressed as mean ± SEM; n = 5 mice per group, three brain sections analyzed per mouse. *P < 0.05, **P < 0.01 versus the Saline group by two-tailed independent samples t-test.

### 3.3 The TRPC5 inhibitor AC1903 alleviates NTG-evoked migraine-like behavioral abnormalities

Pharmacological intervention with AC1903 was performed to explore the functional relevance of TRPC5 (**Figure 3A**). Migraine-like phenotypes were evaluated across light-aversive-like behavior, thermal nociceptive hypersensitivity and mechanical nociceptive hypersensitivity. Considering the known involvement of TRPC5 in anxiety regulation, the objective term “light-aversive-like behavior” was adopted instead of “photophobia” to avoid interpretive bias. NTG induced prominent light-aversive-like responses, elevated thermal nociceptive sensitivity and reduced mechanical withdrawal thresholds. AC1903 treatment effectively reversed these abnormalities, improved light-aversive-like behaviors under both acute and chronic paradigms (**Figures 3B, C**), alleviated thermal nociceptive hypersensitivity-like phenotypes (**Figures 3D, E**), and restored mechanical withdrawal thresholds (**Figures 3F, G**). These behavioral results indicate that TRPC5 activity correlates significantly with NTG-triggered migraine-like behavioral alterations.

**Figure 3:**
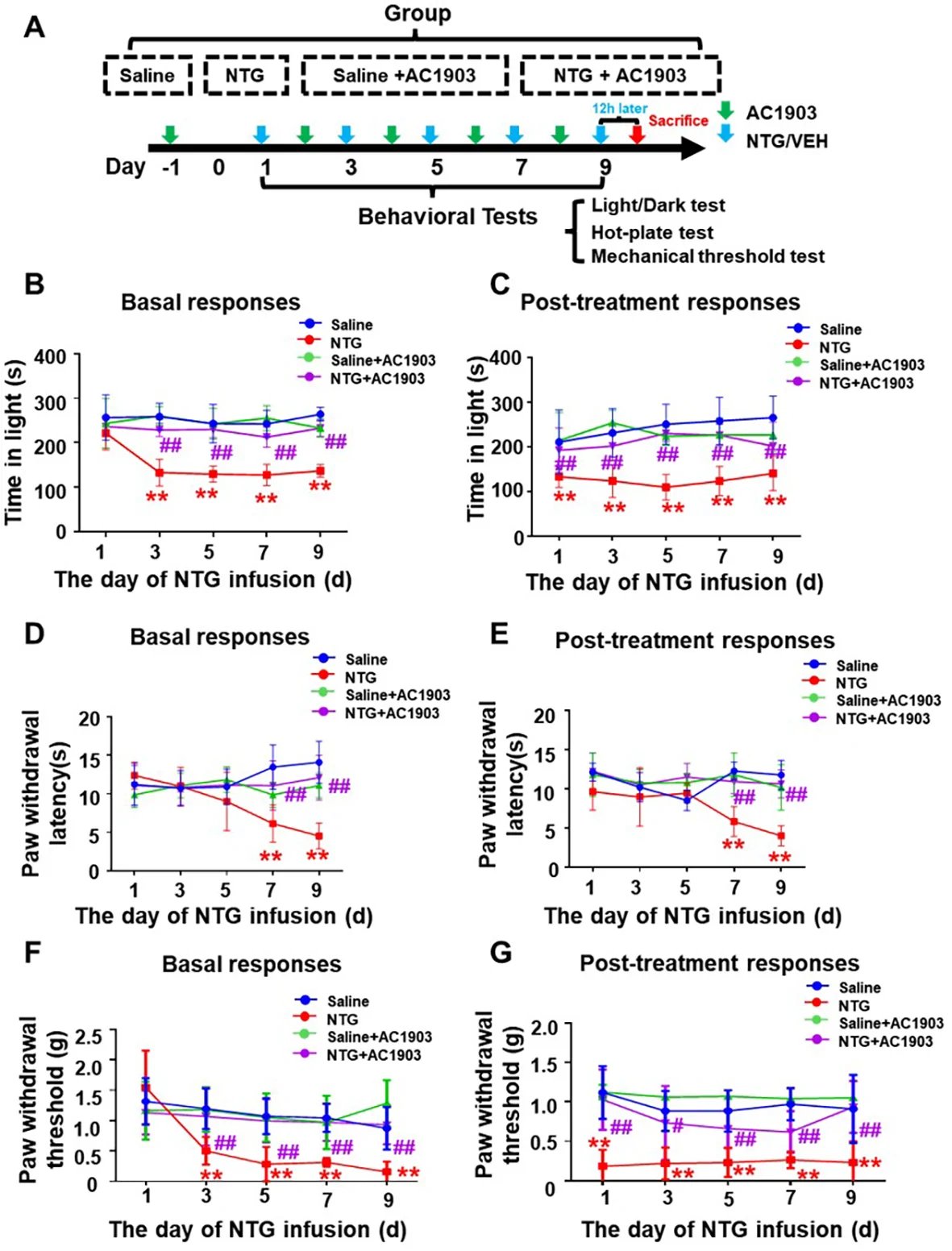
The TRPC5 antagonist AC1903 reverses NTG-mediated reduction of pain thresholds in migraine models. **(A)** Schematic paradigm of AC1903 administration for pain threshold measurement in NTG-induced migraine mice. **(B, C)** AC1903 ameliorated photophobia in mice with migraine-like phenotypes under chronic **(B)** and acute **(C)** migraine conditions. **(D, E)** AC1903 relieved thermal hyperalgesia in acute **(E)** and chronic **(D)** migraine mice. **(F, G)** Mechanical pain thresholds were restored by AC1903 in both chronic **(F)** and acute **(G)** migraine mice. Data are presented as mean ± SEM; n = 8 per group; two-way ANOVA and Tukey *post hoc* analysis; **P<0.01 compared to the Saline group, ##P < 0.01 compared to the NTG group, by two-way ANOVA and Tukey *post hoc* test.

### 3.4 AC1903 suppresses cellular activation and attenuates PI3K-Akt pathway activity in the SSP cortex

c-Fos staining revealed that NTG increased c-Fos-positive cell counts in the SSP cortex, indicating robust cellular activation. AC1903 reduced c-Fos-positive cell numbers and the c-Fos-positive/NeuN-positive ratio, mitigating NTG-induced cellular hyperactivation (**Figures 4A–C**). It should be emphasized that c-Fos is not neuron-specific; c-Fos-positive cells represent overall activated cortical cell populations rather than exclusively activated neurons. Further Western blot detection of core PI3K-Akt signaling proteins in SSP cortex (**Figures 4D–F**) showed that NTG increased phosphorylation of PI3K and Akt, which was effectively blunted by AC1903. These data suggest that elevated TRPC5 activity correlates with excessive PI3K-Akt activation and cortical cellular hyperactivation.

**Figure 4:**
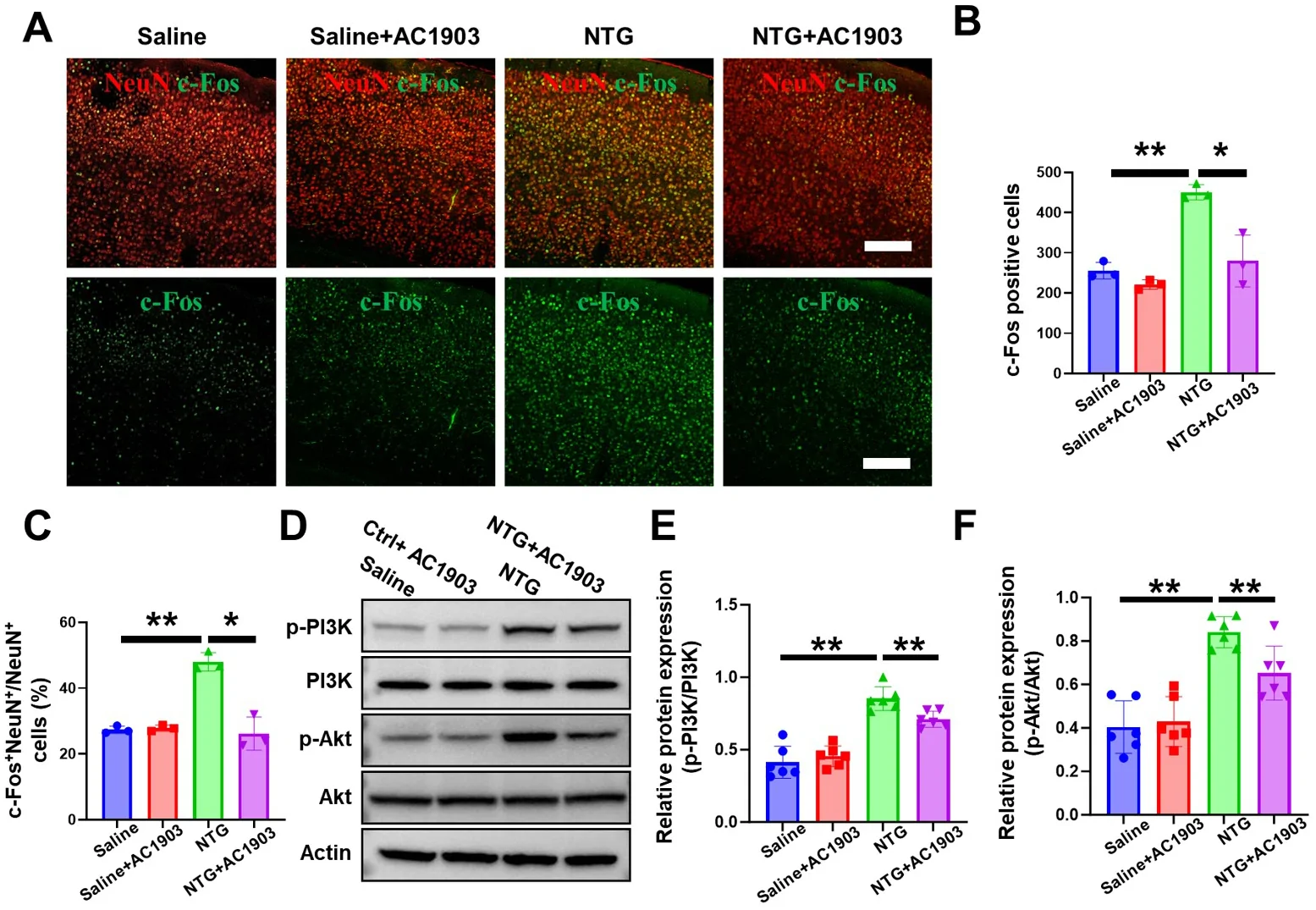
AC1903 suppresses NTG-triggered neuronal activation and antagonizes hyperactivation of the PI3K-Akt signaling pathway in the SSP region. **(A)** Representative immunofluorescence images showing NTG-induced neuronal activation in the SSP region, which was effectively reversed by AC1903 treatment. Scale bar: 100 μm. **(B, C)** Quantitative analysis demonstrated that AC1903 markedly reduced NTG-evoked increases in the number of c-Fos^+^ neurons **(B)** and the proportion of c-Fos^+^/NeuN^+^ neurons among total NeuN^+^ neurons **(C)**. **(D)** Representative western blot images of core protein expression within the PI3K-Akt signaling pathway in different experimental groups. **(E, F)** AC1903 significantly inhibited NTG-induced upregulation of PI3K **(E)** and Akt **(F)**. Data are presented as mean ± SEM; n = 3 per group, and 3 brain sections were analyzed per mouse in the immunofluorescence assay; n = 6 per group in the WB assay. *P < 0.05, **P < 0.01 versus the Saline or NTG group, Two-tailed independent-samples t-test; one-way ANOVA with Tukey’s *post hoc* test for multiple-group comparisons.

### 3.5 TRPC5 inhibition ameliorates NTG-driven microglial activation and elevated neuroinflammatory-related molecules

Iba1 immunofluorescence was applied to assess microglial activation status in SSP cortex (**Figure 5A**). NTG induced robust microglial activation, which was morphologically characterized by increased cell number, shortened processes, and enlarged somata. AC1903 reversed these morphological alterations: it increased the total and average process lengths of Iba1-positive microglia, reduced the counts of Iba1-positive microglia, and decreased the soma area fraction of activated microglia. (**Figures 5B–E**). Western blot and qPCR demonstrated that NTG elevated P2X7R and IL-1β, and AC1903 significantly suppressed both molecules (**Figures 5F–H**). Notably, only total cortical tissue lysates were assayed. Co-localization for P2X7R and IL-1β with glial or neuronal markers was not performed; therefore, cellular sources of these proteins cannot be distinguished. We can conclude that TRPC5 inhibition correlates with reduced microglia activation and inflammatory factor levels.

**Figure 5:**
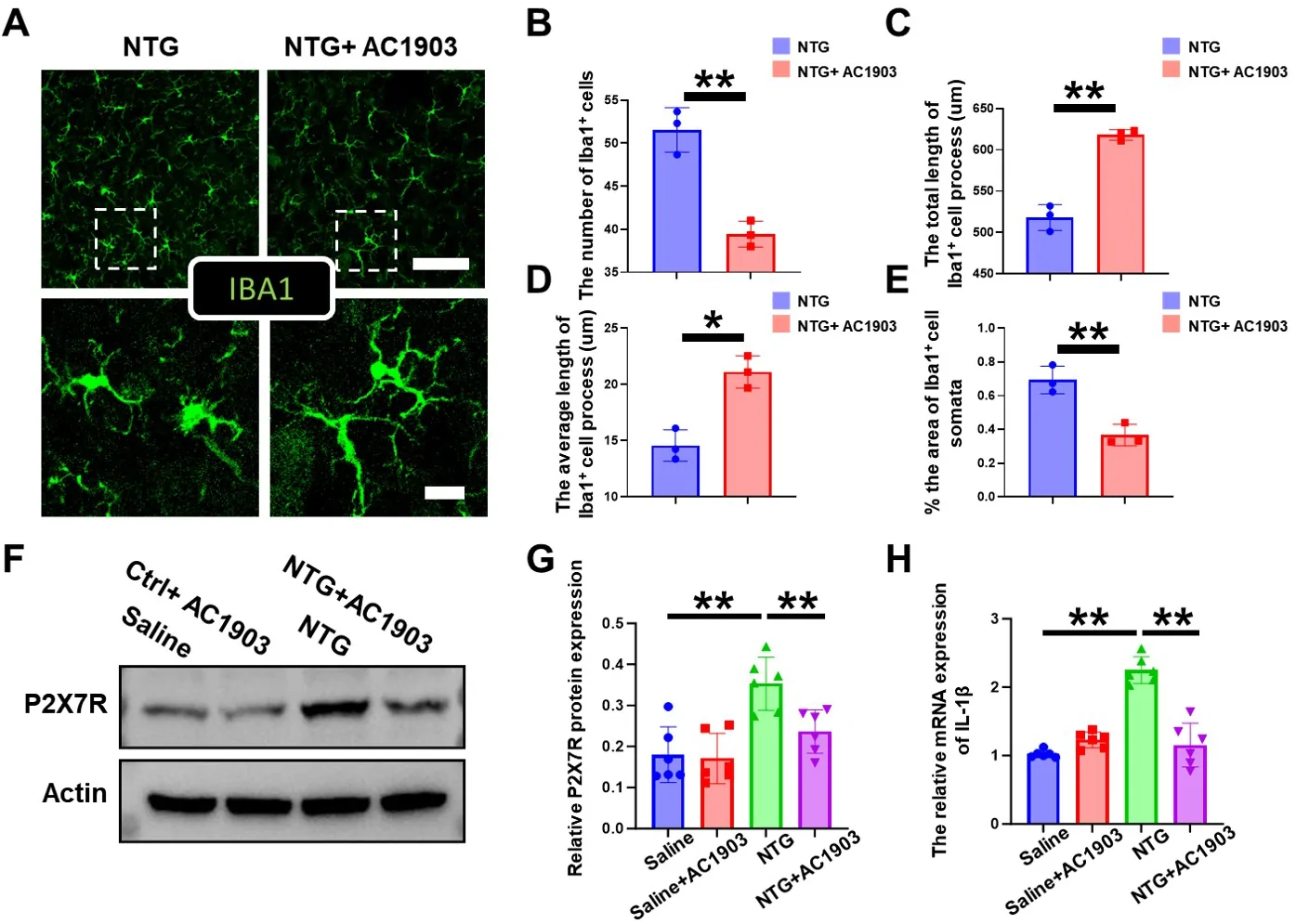
AC1903 suppresses NTG-triggered microglial activation as well as the upregulation of P2RX7 and IL-1β within the SSP region. **(A)** Representative immunofluorescence images demonstrate NTG-mediated microglial activation in the SSP region, which is reversed by AC1903 treatment. Scale bars: 100 μm (upper images), 10 μm (lower images). **(B–E)** Quantitative analysis of microglial morphological parameters in the NTG group and NTG+AC1903 group, including the count of Iba1^+^ microglia **(B)**, total length of Iba1^+^ cellular processes (μm) **(C)**, average process length per microglia (μm) **(D)**, and the area fraction of Iba1^+^ somata **(E)**. **(F–H)** Representative western blot bands **(F)** and quantitative statistical results for P2RX7 protein expression **(G)** and IL-1β **(H)** mRNA expression level in each group. Data are expressed as mean ± SEM; n = 3 per group for the immunofluorescence assay and n = 6 per group for the WB and qPCR assay. *P < 0.05, **P < 0.01 versus the Saline or NTG group. Two-tailed independent-samples t-test; one-way ANOVA with Tukey’s *post hoc* test for multiple-group comparisons.

### 3.6 The PI3K-Akt inhibitor LY294002 attenuates NTG-induced migraine-like behaviors

The non-selective PI3K inhibitor LY294002 was used to block the PI3K-Akt pathway and investigate its potential role in migraine-like central sensitization (**Figure 6A**). LY294002 ameliorated acute- and chronic-phase light-aversive-like behavioral abnormalities induced by NTG (**Figures 6B, C**), reversed NTG-induced reduction of mechanical withdrawal thresholds and alleviated nociceptive hypersensitivity-like behaviors (**Figures 6D, E**). These results suggest that excessive PI3K-Akt activation participates in NTG-provoked migraine-like behaviors and central sensitization.

**Figure 6:**
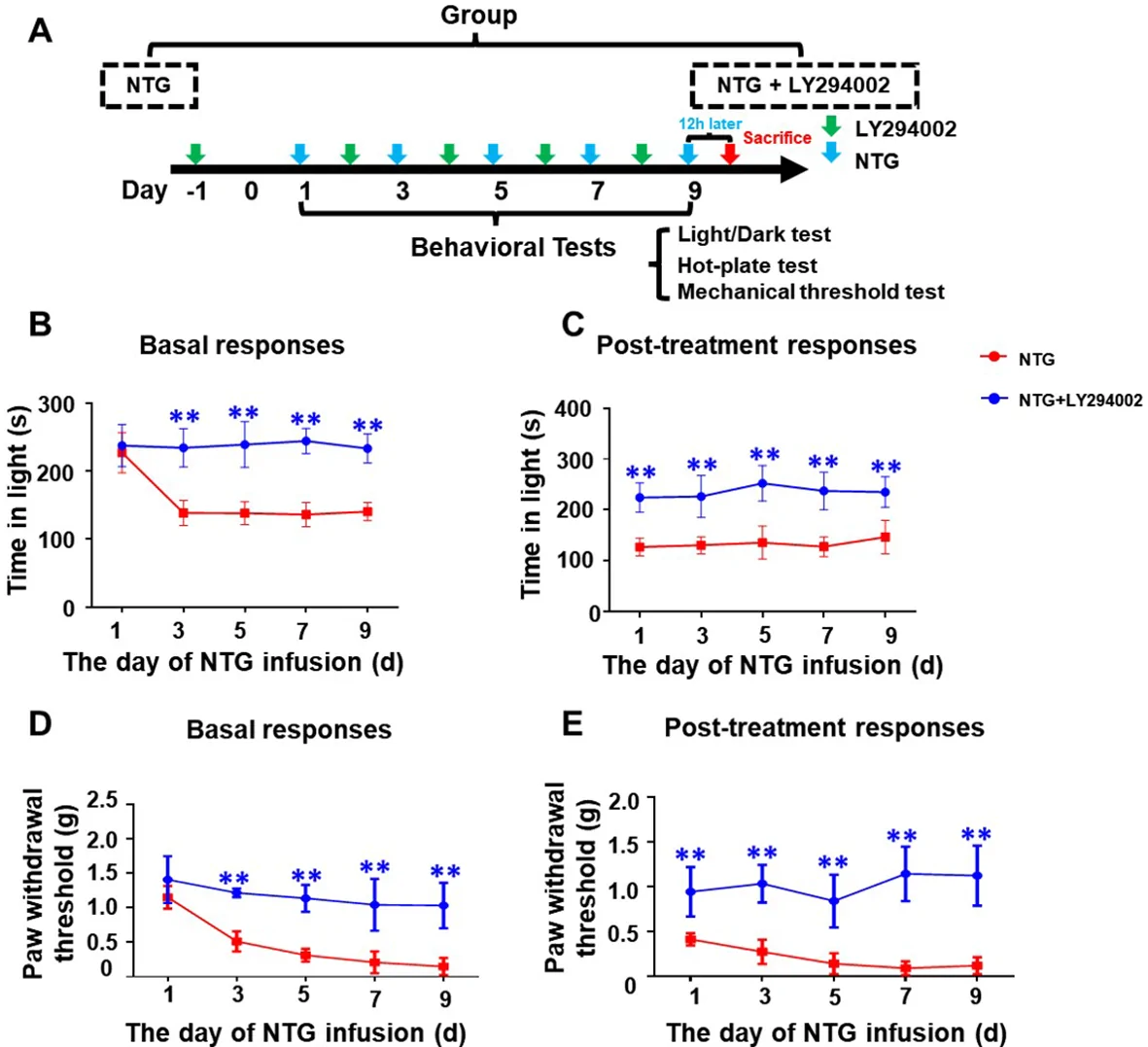
Experimental design and behavioral testing results following LY294002 intervention in NTG-induced migraine mouse models. **(A)** Schematic diagram illustrating the LY294002 intervention protocol for pain threshold detection in NTG-evoked migraine mice. **(B, C)** LY294002 relieved photophobia in mice with migraine-like phenotypes under chronic **(B)** and acute **(C)** migraine states. **(D, E)** Mechanical pain hypersensitivity triggered by migraine was attenuated by LY294002 in chronic **(D)** and acute **(E)** migraine models, as determined via von Frey filament testing. Data are expressed as mean ± SEM, n = 8 per group. Statistical analysis was performed using two-way ANOVA followed by Tukey *post hoc* multiple comparisons. **P < 0.01 vs. NTG group, by two-way ANOVA and Tukey *post hoc* test.

### 3.7 PI3K-Akt inhibition attenuates cortical cellular activation and neuroinflammatory responses

C-Fos immunofluorescence showed that LY294002 decreased c-Fos-positive cell counts and the proportion of c-Fos-positive neurons, relieving cellular hyperactivation (**Figures 7A–C**). Morphological analysis of Iba1 staining demonstrated that LY294002 restrained NTG-induced microglial over-activation and restored aberrant microglial proliferation and process remodeling (**Figures 7D–H**). Meanwhile, LY294002 suppressed NTG-elevated P2X7R protein level and IL-1β mRNA expression levels (**Figures 7I, J**). Nevertheless, LY294002 is a non-specific inhibitor with off-target risks and limited blood-brain-barrier permeability. These results only support the involvement of the PI3K-Akt pathway in this pathological process and cannot confirm absolute specific regulation. Collectively, pharmacological blockade of PI3K-Akt simultaneously mitigates cortical cellular activation, pathological microglial reactivity and neuroinflammation, indicating that PI3K-Akt hyper-activation constitutes an important correlative component of migraine-like central sensitization.

**Figure 7:**
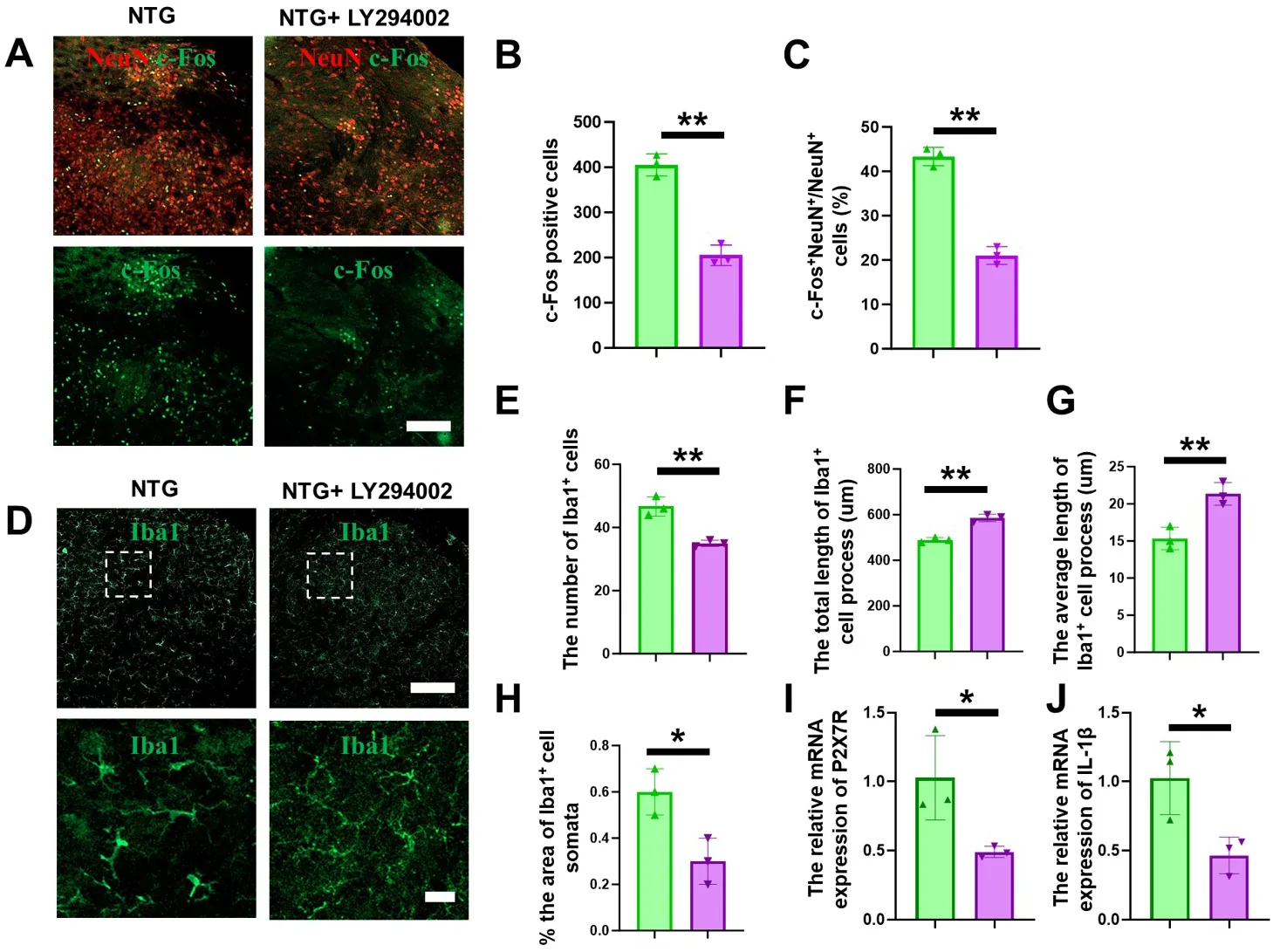
The PI3K-Akt pathway mediates neuronal and microglial activation within the SSP region in NTG-induced migraine mice. **(A)** Representative immunofluorescence images showing neuronal activation in the SSP region triggered by NTG, which was reversed by LY294002 administration. Scale bar = 100 μm. **(B, C)** Quantitative statistics revealed that LY294002 decreased NTG-elicited c-Fos^+^ neuronal counts **(B)** and the ratio of c-Fos^+^NeuN^+^ neurons to total NeuN^+^ neurons **(C)**. **(D)** Representative immunofluorescence staining depicting NTG-mediated microglial activation in the SSP region; this phenotype was counteracted by LY294002. Scale bars: 100 μm (upper panels), 10 μm (lower panels). **(E–J)** Quantitative analyses of microglial morphological parameters between the NTG group and NTG+LY294002 group, including the number of Iba1^+^ microglia **(E)**, total length of Iba1^+^ processes (μm) **(F)**, average process length per microglia (μm) **(G)**, and the area fraction of Iba1^+^ cell somata **(H)**. **(I, J)** Quantitative detection of P2RX7 **(I)** and IL-1β **(J)** expression demonstrated that LY294002 suppressed NTG-induced upregulation of these two genes. Data are expressed as mean ± SEM, n = 3 per group, 2–3 brain sections analyzed per mouse in the immunofluorescence assay. *P < 0.05, **P < 0.01 versus the NTG group, by two-tailed independent samples t-test.

### 3.8 Schematic summary of the working hypothesis

Based on all correlative pharmacological findings in this study, we propose a hypothetical mechanistic model (**Figure 8**). NTG stimulation increases neuronal TRPC5 within the SSP cortex. Increased TRPC5 correlates with excessive PI3K-Akt activation and contributes to neuronal hyper-excitability. Neuronal hyperactivation is accompanied by microglial reactivity and up-regulation of the P2X7R/IL-1β inflammatory axis, jointly contributing to NTG-evoked migraine-like central sensitization.

**Figure 8:**
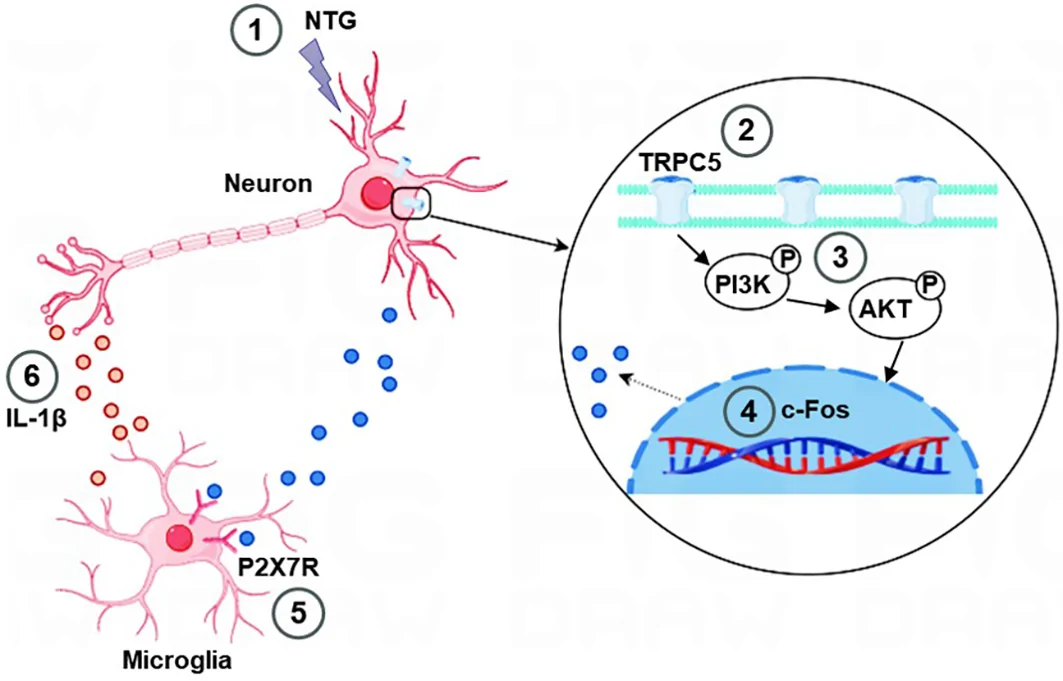
Schematic illustration of the molecular mechanism proposed in this study. ① ② NTG stimulation upregulates TRPC5 expression; ③ elevated TRPC5 further triggers activation of the PI3K-Akt signaling pathway; ④ persistent PI3K-Akt signaling drives neuronal activation; ⑤ activated neurons initiate microglial activation through P2X7 receptors; ⑥ reactive microglia secrete pro-inflammatory cytokine IL-1β, ultimately facilitating migraine central sensitization.

## 4 Discussion

Using the NTG-induced mouse migraine-like model, the present study explored the potential role of TRPC5 in migraine-associated central sensitization. We observed increased TRPC5 mRNA and protein abundance in the SSP cortex of migraine-model mice. Immunofluorescence co-localization indicated that TRPC5 elevation occurs predominantly in NeuN-positive neurons. *In-vivo* pharmacological blockade of TRPC5 with AC1903 improved migraine-like behaviors, reduced cortical cellular hyperactivation, mitigated microglial reactivity and down-regulated P2X7R and IL-1β expression levels. Combined with results obtained from the pathway inhibitor LY294002, our correlative data suggest that TRPC5 may participate in migraine-like central sensitization via the PI3K-Akt signaling axis and is associated with P2X7R/IL-1β-mediated neuroinflammation.

Chronic migraine represents a highly-disabling neurological disorder characterized by recurrent headache accompanied by diverse sensory disturbances, substantially impairing patients’ quality of life and social functioning (10, 26). Cutaneous allodynia serves as a prominent clinical manifestation of underlying central sensitization (26). NTG is a well-established migraine trigger; repeated intraperitoneal NTG injection in rodents recapitulates migraine-related nociceptive hypersensitivity phenotypes (27, 28). Repeated NTG exposure reduces mechanical withdrawal thresholds in mice, mimicking craniofacial and extracranial allodynia observed in migraine patients (29, 30). Migraine exhibits higher prevalence among female humans linked to estrogen fluctuation (31), yet stable migraine-like phenotypes can be induced in both male and female rodents. To minimize estrogen-related confounding effects, only male mice were used in this study. Due to this experimental limitation, our findings cannot be directly generalized to female subjects. Further investigations are required to evaluate potential sex-dependent effects of TRPC5 in chronic migraine.

TRPC5 is a high-calcium-permeable non-selective cation channel. Functional tetrameric channels mediate Ca²^+^ and cation influx and modulate intracellular calcium homeostasis (15). Recent cryo-EM studies have resolved high-resolution TRPC5 structures, advancing understanding of channel gating and regulatory mechanisms (32). TRPC5 was initially identified within the central nervous system, with markedly higher expression in brain tissue relative to most peripheral tissues (33). Cumulative evidence implicates TRPC5 in diverse pain-related pathological conditions, yet its expression and functional outputs vary across different pain models. Synovial TRPC5 is down-regulated in osteoarthritis and rheumatoid arthritis (34, 35), whereas TRPC5 is up-regulated in inflamed dental pulp and spinal cord after chronic morphine exposure (18, 36). Pre-clinical animal experiments indicate that TRPC5 hyper-activation contributes to mechanical nociceptive hypersensitivity and persistent spontaneous pain in certain models, and pharmacological TRPC5 inhibition yields analgesic effects (37). Consistent with these prior reports, we detected elevated SSP-cortical TRPC5 in NTG-treated mice, and AC1903 ameliorated migraine-like behaviors, supporting a correlation between altered TRPC5 expression and NTG-evoked nociceptive responses.

Central sensitization constitutes a core pathological basis of chronic migraine, clinically manifested as extracranial and intracranial allodynia and expanded pain receptive fields (3, 29). Repeated NTG administration induced nociceptive hypersensitivity in mice, confirming successful modeling of migraine-like central sensitization. The immediate-early gene c-Fos is widely adopted as a marker of noxious-stimulus-triggered cellular activation (38, 39). Nevertheless, c-Fos is not neuron-specific and reflects overall tissue cellular activation status. Increased c-Fos-positive cell counts were observed in the SSP cortex of NTG-model mice, and AC1903 treatment lowered cellular activation levels, further supporting a correlation between TRPC5 activity and cortical cellular hyper-reactivity.

The PI3K-Akt pathway modulates neuronal synaptic plasticity, dendritic spine remodeling and synaptic transmission efficacy and plays critical roles in pain-associated central sensitization (20, 21). To dissect downstream molecular events linking TRPC5 to migraine-like sensitization, we assessed PI3K-Akt phosphorylation status. TRPC5 inhibition attenuated NTG-induced hyper-phosphorylation of PI3K and Akt, indicating functional crosstalk between TRPC5 and PI3K-Akt activation. PI3K-Akt activation enhances NF-κB transcriptional activity, promoting transcription and secretion of pro-inflammatory mediators including IL-1β (40). Released pro-inflammatory cytokines further drive microglial activation, forming inflammatory positive-feedback loops that amplify central sensitization (23). Previous studies have documented microglial hyper-reactivity in chronic-migraine models, featuring increased cell numbers, soma hypertrophy and process remodeling (41, 42).

Upon stimulation, neurons release ATP, which acts on microglial P2X and P2Y purinergic receptors. Dysregulated purinergic receptor signaling contributes to migraine-related central sensitization (43). Animal studies indicate that P2X7R deletion reduces c-Fos expression and improves migraine-like behaviors, suggesting that P2X7R may mediate neuron-microglia communication and serve as a potential therapeutic target for migraine (44). In our study, TRPC5 inhibition was accompanied by reduced P2X7R and IL-1β abundance.

Collectively, this study provides pre-clinical experimental evidence demonstrating that up-regulated SSP-cortical TRPC5 correlates with NTG-evoked migraine-like central sensitization in mice. Pharmacological TRPC5 inhibition ameliorates migraine-like behaviors, suppresses PI3K-Akt pathway activity, and mitigates cortical cellular hyperactivation, pathological microglial morphology and elevated P2X7R/IL-1β. Our correlative results suggest TRPC5 as a potential therapeutic target for chronic migraine. Nevertheless, definitive molecular regulatory mechanisms await further *in-vitro* and *in-vivo* validation.

## 5 Limitations of the study

Several limitations should be acknowledged. First, male mice were exclusively used to eliminate estrogen-dependent biological confounders. Estrogen fluctuations in female individuals can significantly modulate pain sensitivity and TRPC5 channel activity; therefore, our findings cannot be directly generalized to female migraine patients. Given the higher clinical prevalence of migraine among females, potential sex-dependent TRPC5-mediated migraine pathogenesis requires further validation in female animal models to improve the generalizability of our conclusions. Second, we adopted the NTG mouse model, which recapitulates acute migraine attacks and partial central sensitization phenotypes, but cannot fully reproduce all pathological features of human chronic migraine and fails to mimic the disease progression from episodic to chronic migraine. Third, this study primarily relies on pharmacological TRPC5 inhibition, which may exert off-target effects on other ion channels. Therefore, the conclusions drawn from pharmacological intervention should be interpreted with caution. Genetic knockout or overexpression animal models are required to confirm the direct and specific functions of TRPC5 during migraine progression. Fourth, although we characterize regulatory relationships among the PI3K-Akt, P2X7R and IL-1β signaling axis, upstream mechanisms triggering TRPC5 activation during migraine pathogenesis remain incompletely clarified. Finally, we only conducted TRPC5-NeuN co-immunofluorescence staining to identify neuronal TRPC5 expression. Further glial co-localization assays are required to clarify the cell-type distribution of TRPC5 in Sp5C. Future clinical investigations are needed to validate the translational potential of TRPC5 as a therapeutic target for human chronic migraine.

## 6 Conclusions

In summary, the present study suggests that TRPC5 participates in pathological processes ranging from acute onset to chronic progression in NTG-induced mouse migraine models, correlating with neuronal hyperactivation, neuron-microglia crosstalk and central sensitization. TRPC5 expression is increased in SSP-cortical neurons of NTG-model mice, accompanied by neuronal hyperexcitability and persistent nociceptive hypersensitivity. The selective TRPC5 antagonist AC1903 alleviates acute migraine-like sensory abnormalities, suppresses neuronal hyperactivation, and improves central-sensitization-related phenotypes under both acute and chronic experimental paradigms.

Based on our pharmacological results, we propose a working hypothesis: elevated TRPC5 contributes to excessive PI3K-Akt activation, which may facilitate ATP release and P2X7R up-regulation, trigger microglial activation and IL-1β secretion, and establish inflammatory positive-feedback loops participating in acute nociceptive responses and maintenance of chronic central sensitization.

Our pre-clinical data indicate TRPC5 as a potential upstream modulator of migraine-related central sensitization, possibly acting via the PI3K-Akt/P2X7R-IL-1β signaling axis, offering candidate directions for therapeutic intervention against acute and chronic migraine. This study mainly relies on pharmacological manipulations; causal evidence from genetic editing and direct ATP quantification is lacking, and definitive mechanisms remain to be further verified.

Finally, In the present study, we selected the SSP cortex instead of Sp5C, because SSP serves as a key upstream afferent region innervating Sp5C within the trigeminovascular pain network, enabling us to investigate presynaptic inputs to Sp5C in chronic migraine.

## Data Availability

The original contributions presented in the study are included in the article/**Supplementary Material**. Further inquiries can be directed to the corresponding author.
