# Supplementary material for: TRPC5 involvement in NTG-induced migraine-like central sensitization in mice: a study based on the PI3K-Akt/P2X7R-IL-1β signaling axis
Source: Front Immunol. 2026 Oct 29;17:2018949. doi: 10.3389/fimmu.2026.2018949 (PMC13754658; doi:10.3389/fimmu.2026.2018949)
Supplement: Supplementary file 1 [file DataSheet1.pdf]

Fig 1

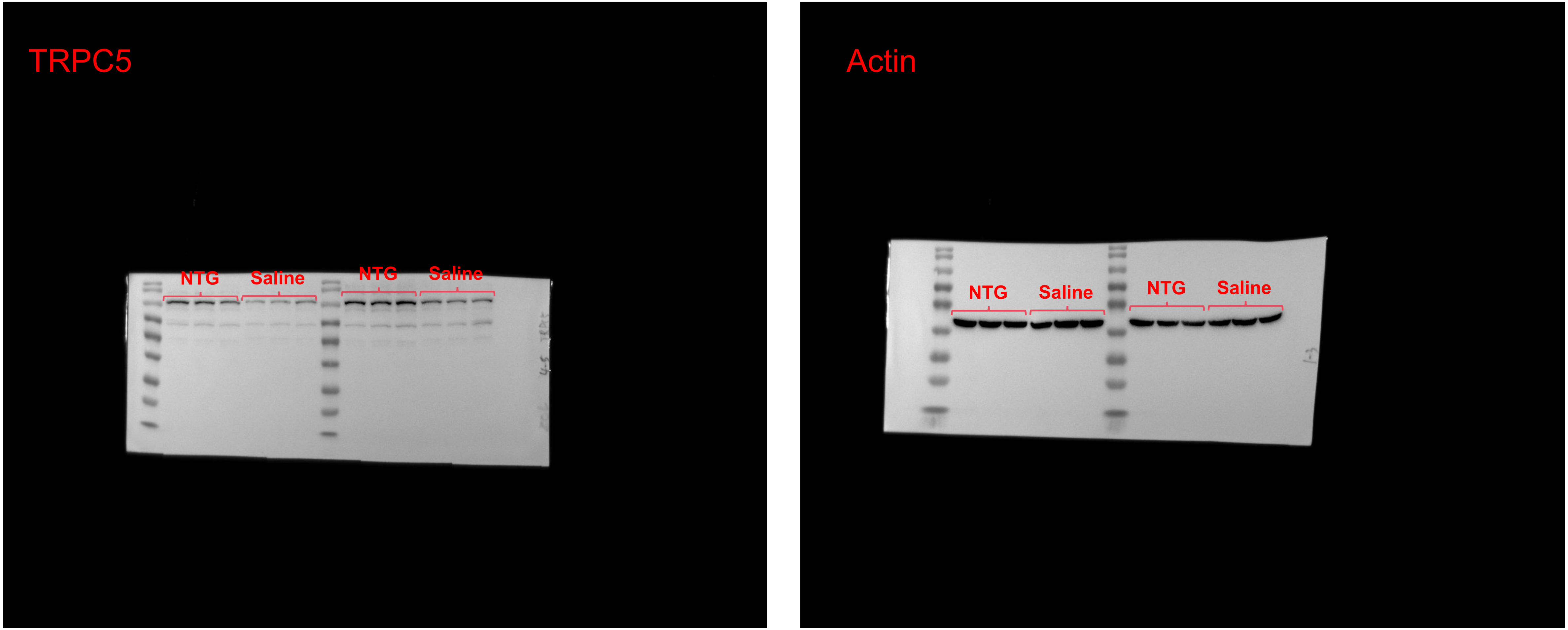

N=6 / group

Fig 4 and 5

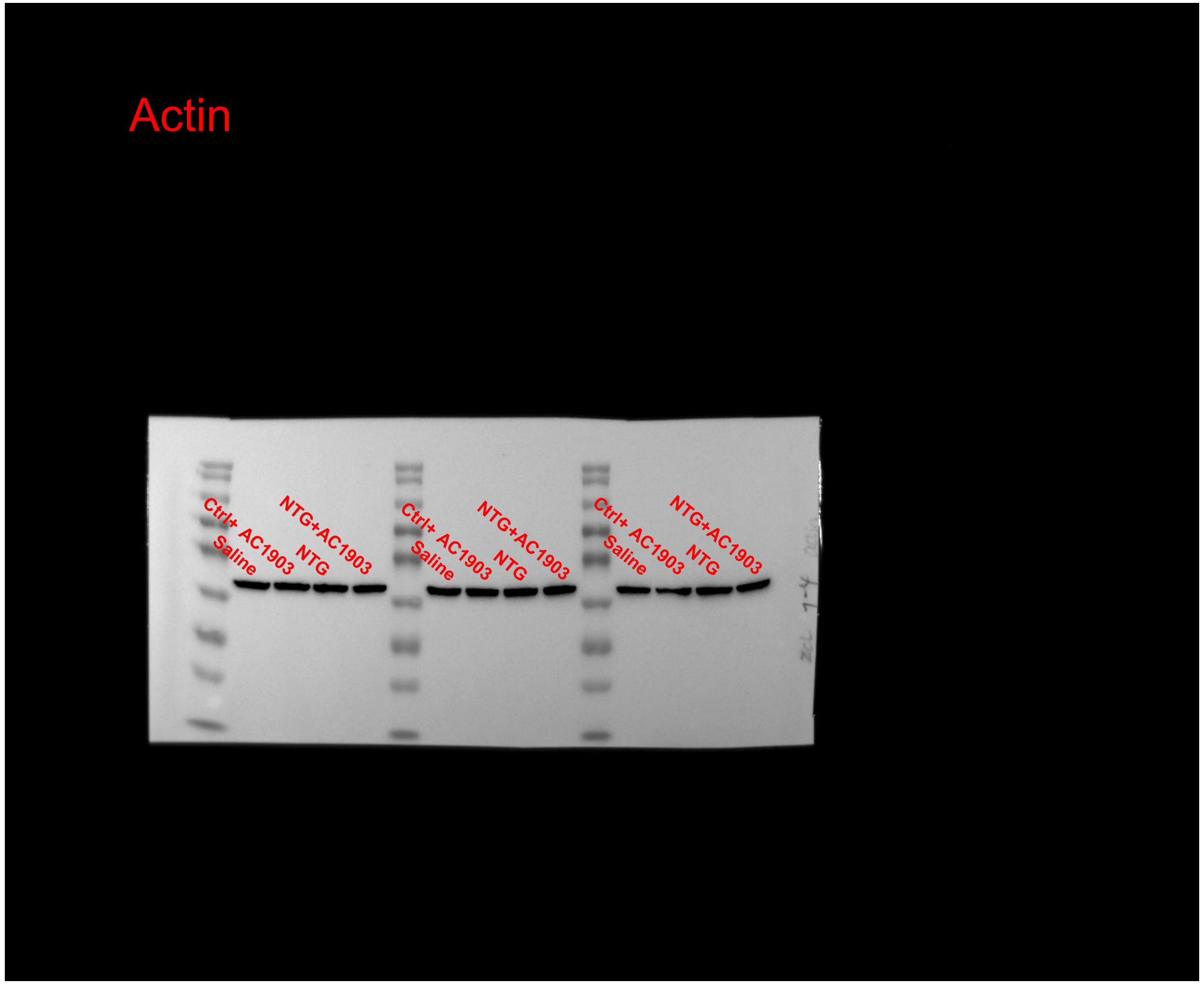

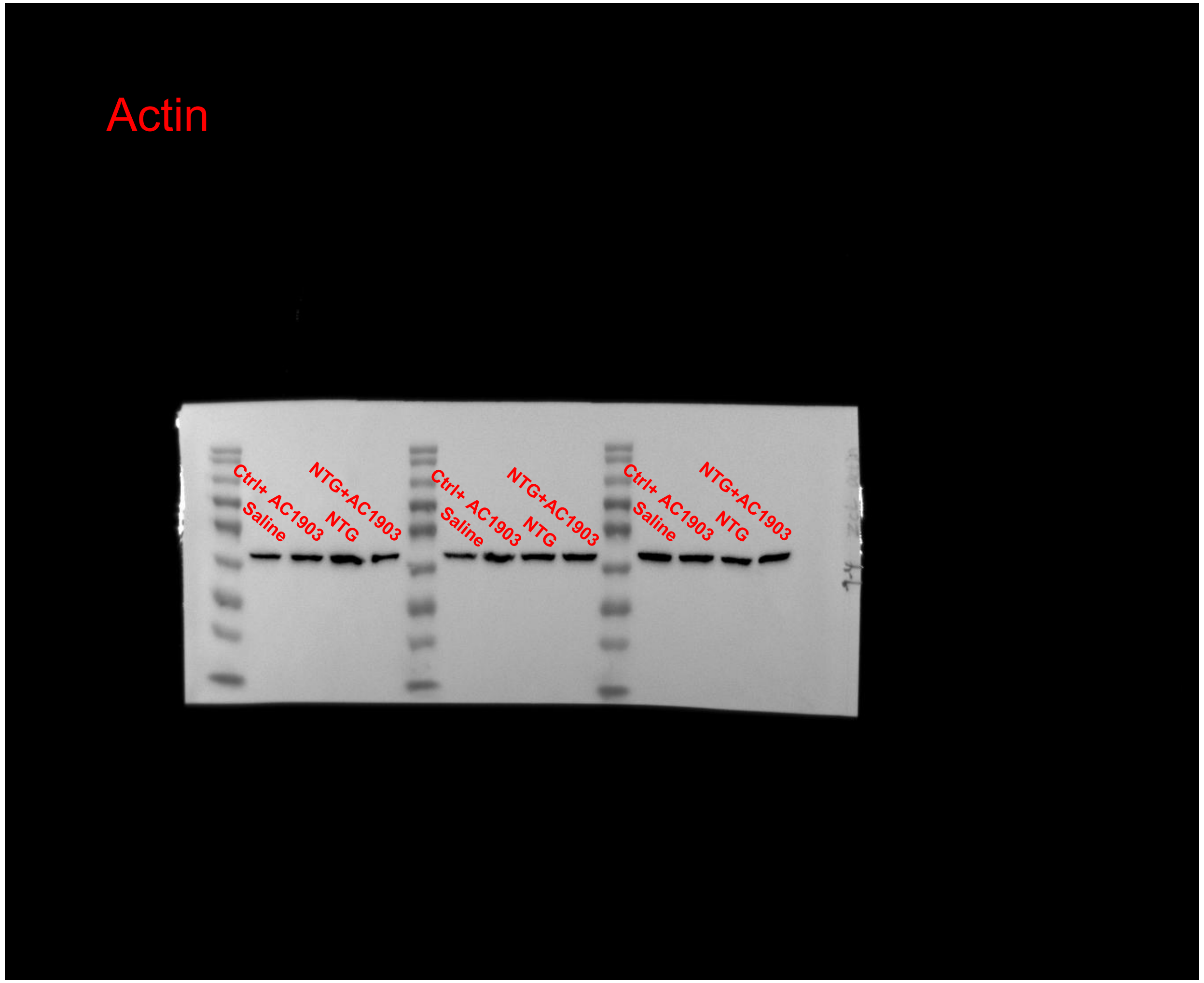

N=6 / group

Fig 4 and 5

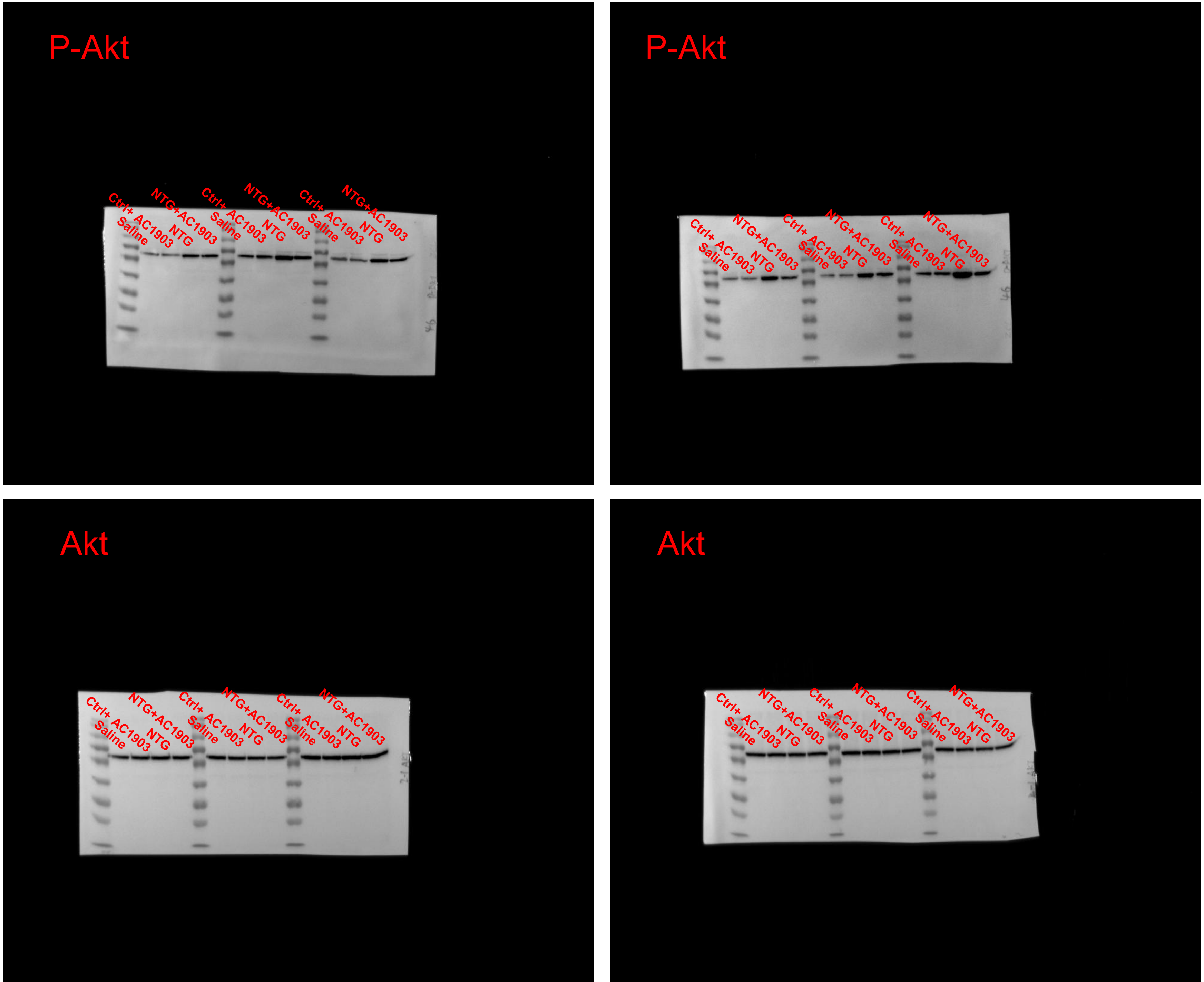

N=6 / group

Fig 4 and 5

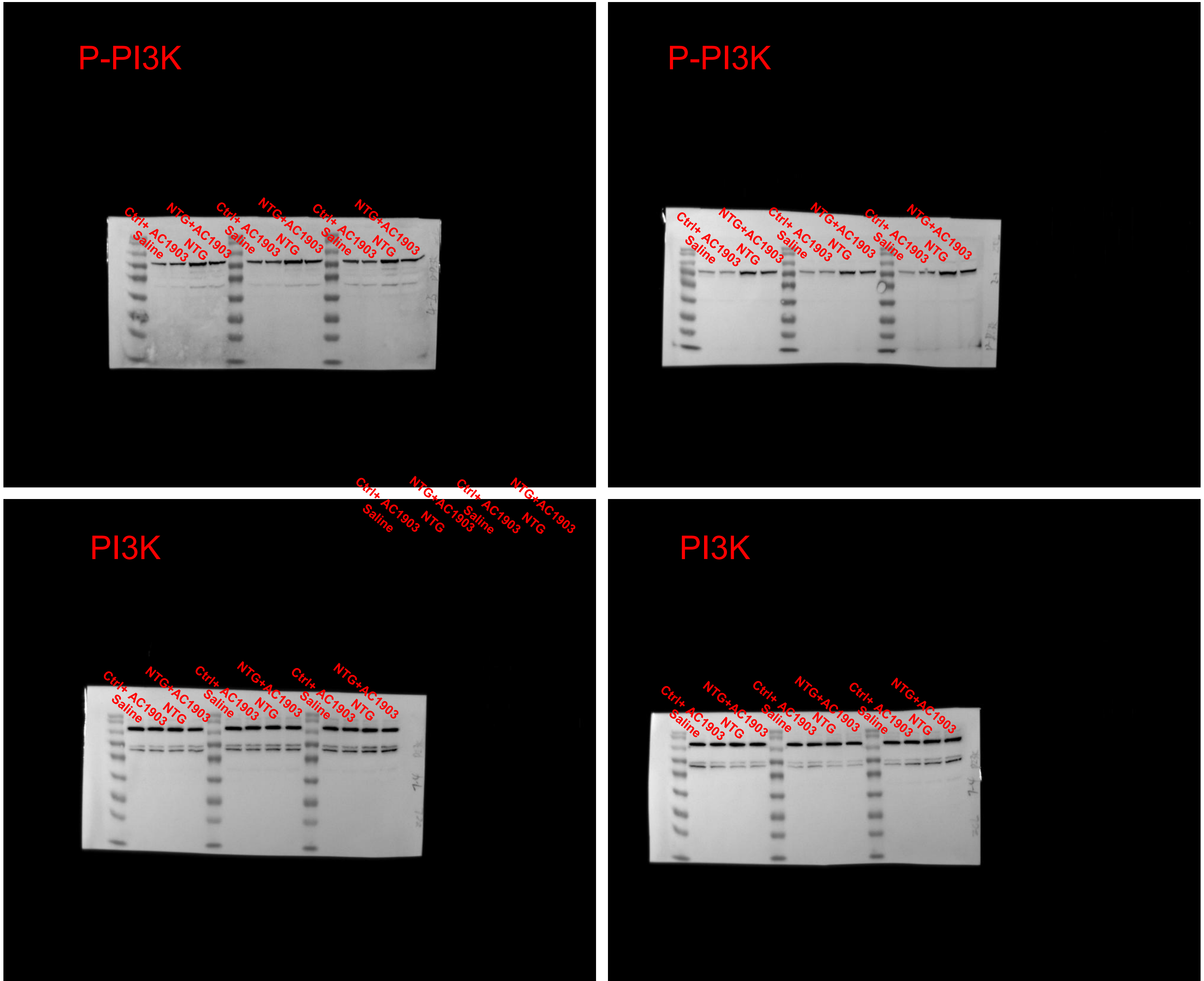

N=6 / group

Fig 4 and 5

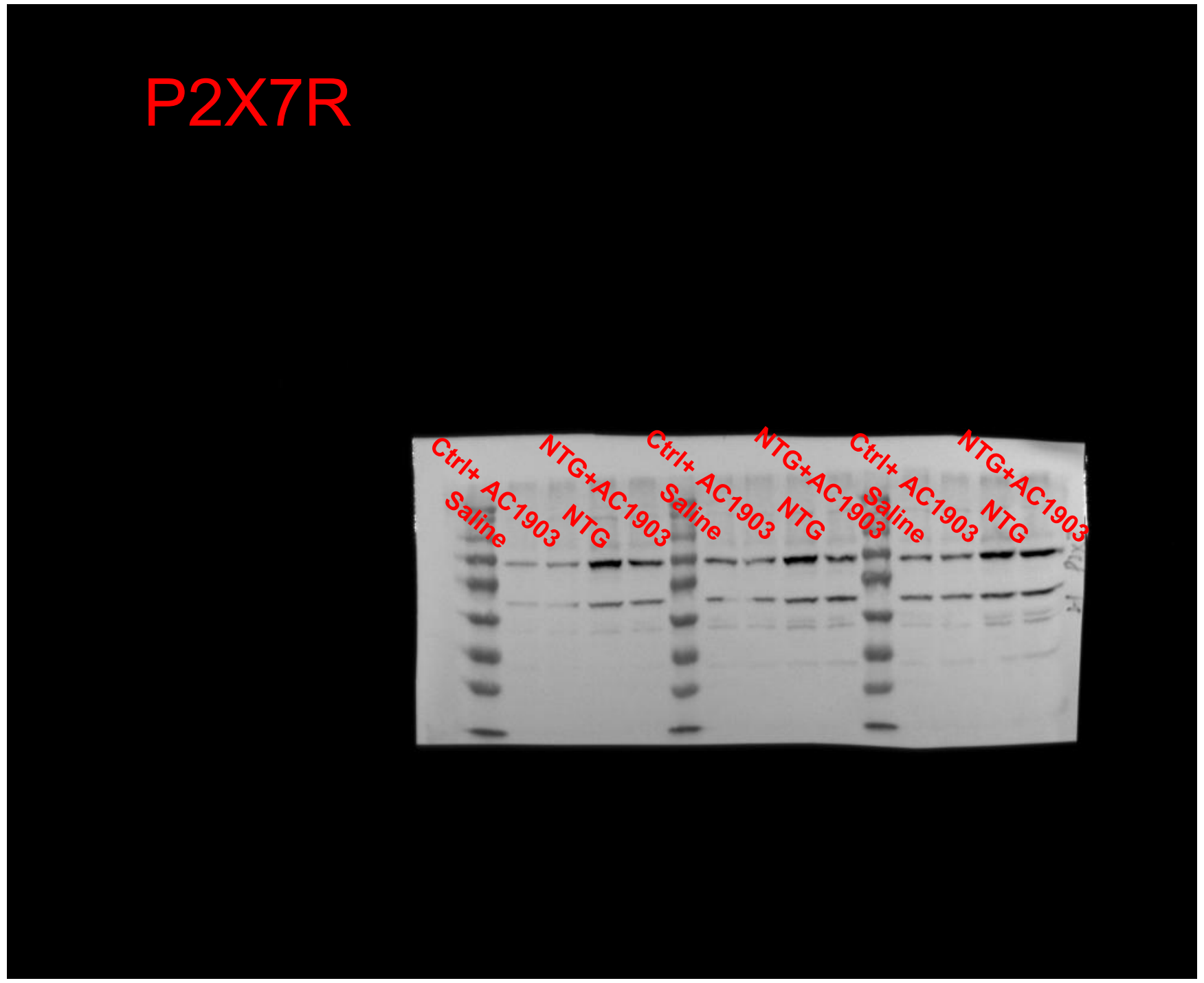

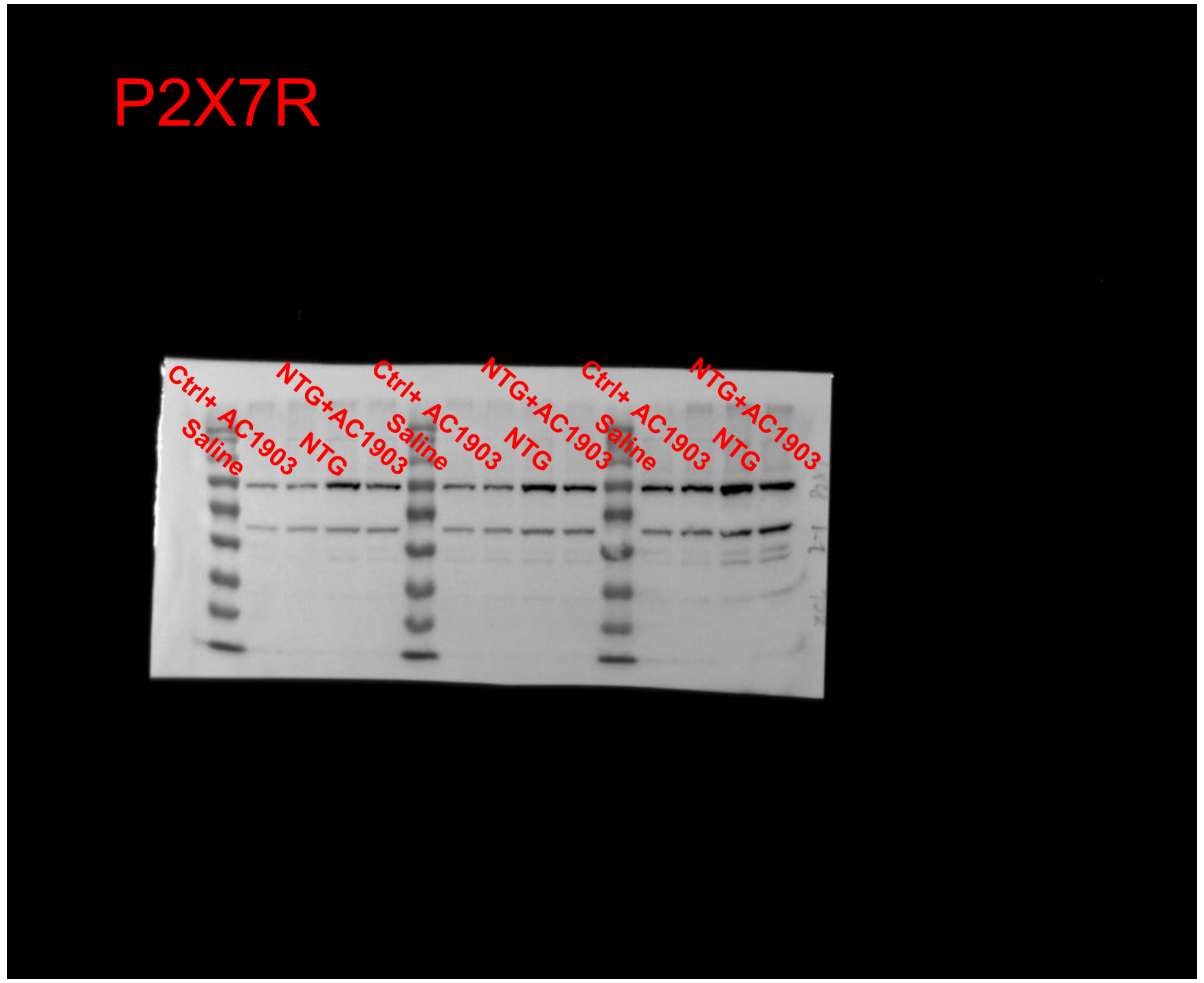

N=6 / group
